# Successful Pregnancy in Autoimmune Hepatitis/Primary Biliary Cirrhosis Overlap Syndrome: A Case Report

**DOI:** 10.4021/gr485w

**Published:** 2012-09-20

**Authors:** Subarna Mitra, Prasanta Kumar Nayak, Alaganandam Padma, George Kurian

**Affiliations:** a Pondicherry Institute Of Medical Sciences, Medical faculty, Obstetrics and Gynecology, Pondicherry, India; b Pondicherry Institute Of Medical Sciences, Medical faculty, Gastroenterology, Pondicherry, India

**Keywords:** Autoimmune hepatitis, Primary biliary cirrhosis, Overlap syndrome

## Abstract

Medical literature on pregnancy outcomes in hepatic overlap syndromes is limited. Autoimmune liver diseases are associated with sub-fertility and pregnancy is usually dissuaded in presence of portal hypertension and cirrhosis due to guarded prognosis for the pregnancy as well as the liver disease. We report a 24-year-old primigravida with autoimmune hepatitis/primary biliary cirrhosis overlap syndrome who had a successful pregnancy owing to intensive monitoring and meticulous treatment with drugs like immunosuppressants and ursodeoxycholic acid. She developed preterm prelabour rupture of membranes and delivered at 35 weeks without any major obstetric or hepatic complications.

## Introduction

Autoimmune hepatitis (AIH), primary biliary cirrhosis (PBC) and primary sclerosing cholangitis (PSC) are the three major autoimmune hepatopathies. The term ‘overlap syndrome’ is used to describe presence of overlapping features of this spectrum of autoimmune disorders, although standard definitions are lacking [1]. AIH/PBC overlap is the commonest, affecting 10% of patients diagnosed with AIH or PBC, both of which have a female preponderance [2, 3]. Usually, women between 40 and 60 years of age are affected. Earlier studies [4, 5] reported a guarded prognosis for pregnancy with AIH and PBC, especially in presence of cirrhosis, but recent scenario is promising. Although there is diverse literature on hepatic overlap syndromes, reports of pregnancy outcomes in this clinical entity are very scarce.

## Case Report

A 24-year-old lady with AIH/PBC overlap syndrome presented to our OPD with positive urine pregnancy test. She had excessive fatigue, right hypochondrial pain, jaundice and pruritus three years ago, but was not evaluated. When these symptoms persisted and additionally, night blindness with bone pain developed, she underwent a detailed work up which revealed raised liver enzymes (SGPT: 47 IU/L, SGOT: 72 IU/L, ALP: 200 IU/L, GGT: 132 IU/L), total bilirubin (6.6 mg/dL) and IgG (2,486 mg%) with positive anti-nuclear(ANA) and anti-mitochondrial antibodies(AMA). Investigations for Wilson’s disease (serum copper and ceruloplasmin), viral hepatitis markers, anti-LKM and anti-SMA antibodies were negative. USG and CT showed chronic liver disease with splenomegaly and multiple perisplenic/peripancreatic adenopathy, normal common bile duct and intrahepatic biliary radicles. Liver biopsy revealed cirrhosis with moderate to focal severe interface hepatitis, focal absence of bile ducts and moderate hepatocanalicular cholestasis. Gastroscopy revealed small esophageal varices. Additionally, DEXA scan of lumbar spine revealed severe osteoporosis in the background of low serum 25 -OH vitamin D level (4 ng/mL).

Based upon these biochemical, serological and histological features, an apt diagnosis of AIH/PBC overlap syndrome had been made and started on azathioprine (AZA), prednisolone and ursodeoxycholic acid (UDCA). Additionally, she received treatment for night blindness and osteoporosis. At antenatal booking, her MELD score was 6 and she was on AZA 75 mg, prednisolone 15 mg, UDCA 600 mg and folic acid. Nuchal translucency was 1.47 mm at 11 weeks. AZA dose was reduced due to leucopenia and finally stopped after 1^st^ trimester. Anomaly scan was normal. Anemia and urinary tract infection (UTI) were treated appropriately. There was no intrauterine growth restriction, gestational hypertension, gestational diabetes or coagulopathy.

She had preterm prelabour rupture of membranes (PPROM) at 35 weeks for which she was induced with misoprostol, with prompt preparedness for possible complications. She delivered a 2.15 kg female baby vaginally without any undue prolongation of labour, fetal distress, postpartum haemorrhage (PPH) or sepsis. Postpartum, prednisolone 20 mg and UDCA 600 mg was restarted to prevent possible relapse. Baby received phototherapy for hyperbilirubinemia of prematurity. Patient was discharged after 1 week with advice for interval copper T insertion.

## Discussion

Overlap syndrome should always be considered once an autoimmune disease has been diagnosed. In AIH/PBC overlap, patients present with clinical, biochemical, serological and histological features of both disorders. Biochemistry shows coexistence of typical hepatitic and cholestatic profiles. IgG is markedly elevated in AIH whereas IgM is predominantly high in PBC. Most authors use the diagnostic criteria proposed by Chazouilleres et al in 1998 [6]. Our patient, too, fulfilled this criteria.

Autoimmune liver diseases (AILDs) are known to be associated with hypothalamic dysfunction, premature ovarian failure, autoimmune thyroiditis, endometriosis, amenorrhoea, anovulatory cycles, subfertility, and repeated pregnancy loss [4, 5]. Moreover, the highly variable and unpredictable behavior of these conditions during pregnancy and the associated maternal and fetal complications led obstetricians and hepatologists to dissuade pregnancy in such women. There are reports of increased risk of preterm delivery (most common), fetal loss before 20 weeks of gestation, preeclampsia, low birth weight, variceal bleeding (most dreaded), LSCS, PPH, hepatic failure and maternal deaths in patients with AIH [4, 7-10]. Course of pregnancy in PBC is also not well understood. While Goh et al [11] observed disease progression in 6 patients in a study of 14 pregnancies with PBC, 2 other studies reported remission with judicious use of UDCA [12, 13].

In our case, despite presence of cirrhosis and portal hypertension, the patient had no menstrual irregularities and conceived spontaneously within 1 year of marriage. Her disease, too, was not adequately controlled preconceptionally. Nevertheless, after review of literature, we counseled her to continue the pregnancy with strict vigilance and meticulous therapy. Apart from prematurity, pregnancy was otherwise uneventful, similar to the case report of Efe et al [14]. Whether the cause for preterm delivery was her liver pathology, drugs or recurrent UTI, is uncertain.

Pregnancy, owing to its immunosuppressive state and hormonal milieu, may modify the course of these autoimmune diseases. High levels of circulating estrogen during pregnancy promote a shift from cytotoxic Th1 to anti-inflammatory Th2 cells, favouring attenuation of disease activity and reduction in immunosuppressant dosage or even discontinuation of AZA without adverse pregnancy outcomes [14, 15]. Nonetheless, antenatal flares can occur infrequently [4, 10]. Exacerbation of the disease may occur with each successive pregnancy [7], emphasizing the importance of disease control before pregnancy, planned pregnancy and contraception. Postpartum exacerbations are usual, even for those who are well controlled in pregnancy [4, 8, 9]. An increase in steroid dose is recommended antepartum if discontinuation of AZA is contemplated or peripartum for anticipated relapses. Flares and poor pregnancy outcomes are associated with poor disease control before pregnancy, absence of therapy, poor compliance to therapy, relapses in previous pregnancies, associated cirrhosis and portal hypertension [7]. Our patient had no antenatal recrudescences or hepatic decompensation and her LFT remained normal with prednisone and UDCA. However, postpartum, her transaminases showed a rising trend so prednisone was stepped up to 20 mg.

Therapy in overlap syndromes is empiric since randomized controlled trials are not available for these rare disorders. Anticholestatic therapy with UDCA (13 - 15 mg/kg) is often combined with immunosuppressive therapy with steroids (0.5 mg/kg) and/or AZA (1 - 1.5mg/kg) for complete biochemical remission and arrest of progression of bridging fibrosis [6, 16]. AZA is a category D drug and crosses placenta. Earlier studies [17] have shown teratogenecity in animals, but recent studies have not validated the same [4, 8, 18]. However, there is a slightly increased risk of prematurity and low birth weight [18]. It is used for its ‘steroidal sparing’ effect in AIH [4]. But American Association for the Study of Liver Diseases (AASLD) recommends discontinuation during pregnancy, if possible, with concomitant increase in steroid dose if needed [19]. Postpartum use is safe [15]. Prednisone is a category C drug without any significant transplacental passage or teratogenecity, hence appears to be the preferred treatment for induction and maintainance of remission in AIH [7]. UDCA is a category B drug and is safe in pregnancy and lactation [12]. Liver transplantation is the definitive cure for end-stage disease.

In our case, there were no congenital malformations despite first trimester exposure to AZA, although there was preterm delivery. Also, pancytopenia improved dramatically after discontinuation suggesting that this side-effect is dose-dependent. Vaginal delivery was allowed as the risk of variceal rupture was minimal. Also, there was no contraindication to the use of misoprostol for induction of labor. Since hormonal contraceptives are contraindicated in presence of liver disease and barrier methods have high failure rates, copper T was chosen as the most appropriate, effective and long term contraceptive method. After delivery, steroid monotherapy, albeit with slightly increased doses, was continued without any rebounds.

### Conclusion

Spontaneous conception and successful pregnancy is now a realistic option for women with AILDs, provided the disease is under remission and cirrhosis and portal hypertension are absent or well compensated. Although there are no clinical predictors for the unpredictable behaviour of these disorders during pregnancy, planned pregnancy and appropriate pharmacotherapy coupled with intensive vigilance during pregnancy and several months postpartum is essential to avoid exacerbations and disease progression. Immunosuppressants and UDCA appear to be safe in pregnancy and lactation when used in low doses and a preemptive increase may be needed to avert postpartum flares. Judicious counseling, optimal preparedness for potentially life-threatening complications and coordinated team work between obstetricians and hepatologists seems prudent in dealing with this challenge.
